# Dietary Acacetin Reduces Airway Hyperresponsiveness and Eosinophil Infiltration by Modulating Eotaxin-1 and Th2 Cytokines in a Mouse Model of Asthma

**DOI:** 10.1155/2012/910520

**Published:** 2012-09-24

**Authors:** Wen-Chung Huang, Chian-Jiun Liou

**Affiliations:** Department of Nursing, Chang Gung University of Science and Technology, 261 Wen-Hwa 1st Road, Kwei-Shan, Taoyuan 333, Taiwan

## Abstract

A previous study found that eosinophil infiltration and Th2 cell recruitment are important causes of chronic lung inflammation in asthma. The plant flavonoid acacetin is known to have an anti-inflammatory effect *in vitro*. This study aims to investigate the anti-inflammatory effect of orally administered acacetin in ovalbumin- (OVA-) sensitized asthmatic mice and its underlying molecular mechanism. BALB/c mice were sensitized by intraperitoneal OVA injection. OVA-sensitized mice were fed acacetin from days 21 to 27. Acacetin treatment attenuated airway hyperresponsiveness and reduced eosinophil infiltration and goblet cell hyperplasia in lung tissue. Additionally, eotaxin-1- and Th2-associated cytokines were inhibited in bronchoalveolar lavage fluid and suppressed the level of OVA-IgE in serum. Human bronchial epithelial (BEAS-2B) cells were used to examine the effect of acacetin on proinflammatory cytokines, chemokines, and cell adhesion molecule production *in vitro*. At the molecular level, acacetin significantly reduced IL-6, IL-8, intercellular adhesion molecule-1, and eotaxin-1 in activated BEAS-2B cells. Acacetin also significantly suppressed the ability of eosinophils to adhere to inflammatory BEAS-2B cells. These results suggest that dietary acacetin may improve asthma symptoms in OVA-sensitized mice.

## 1. Introduction

Asthma is a common allergic inflammatory disease of the respiratory tract [1]. The main symptoms of asthma include chest tightness, hypersecretion of mucus, wheezing, and shortness of breath. Severe attacks of asthma maybe cause airway contraction, which can lead to breathing difficulty, asphyxiation, and death [2].

Recent studies have found that Th2 cells are overactivated and expressed in allergic asthmatic patients [3]. Activated Th2 cells secrete cytokines (including IL-4, IL-5, and IL-13) to induce IgE production, and the proliferation and activation of eosinophils, and promote airway hyperresponsiveness (AHR) in asthmatic patients [4]. In addition, inflammatory tracheal epithelial cells also secrete chemokines to attract more eosinophil infiltration into lung tissue [5]. Hence, the inhibition of Th2 cell activation or reduction of the inflammatory response of tracheal epithelial cells would improve symptoms of asthma.

Asthmatic patients commonly take two kinds of medication: preventative and relief agents [6]. Oral or inhaled steroids are common drugs for the treatment or prevention of asthma. However, steroids have many side effects, and research is underway to find asthma treatment alternatives [7].

Acacetin (5,7-dihydroxy-40-methoxyflavone) is a flavone compound isolated from *Saussurea involucrate* or other members of the Asteraceae family [8]. Recent studies have found that acacetin has anti-inflammatory and antitumor effects [8–10]. Acacetin induces apoptosis in human non-small-cell lung cancer A549 cells and gastric carcinoma AGS cells [11, 12]. Acacetin also inhibits the current IKur efficacy of human atrial myocytes and extends the action potential duration of atrial fibers [13]. In addition, acacetin strongly inhibits the expression of proinflammatory cytokines, iNOS, and cyclooxygenase-2 (COX-2) in LPS-induced RAW 264.7 cells [8]. The mechanisms by which acacetin improves asthma symptoms are unclear.

The purpose of this study is to investigate whether acacetin is able to improve symptoms of asthma, including eosinophil infiltration, AHR, and the presence of proinflammatory and Th2-associated cytokines, in OVA-sensitized mice. We also endeavored to evaluate whether acacetin modulates cytokine and chemokine levels in tracheal epithelial cells, and to understand the role of airway epithelial cells in the migration of eosinophils into the lung.

## 2. Materials and Methods

### 2.1. Animals

Female BALB/c mice (6 to 8 weeks old, approximately 20 g each) were purchased from the National Laboratory Animal Center (Taipei, Taiwan). All animal experiments were performed according to the guidelines of the Animal Care Committee of Chang Gung University of Science and Technology and Chang Gung University. Animals were kept in conventional animal housing under standard conditions at the Animal Center of Chang Gung University.

### 2.2. Sensitization and Drug Treatment of Mice

Mice were immunized by intraperitoneal injection with 50 *μ*g chicken ovalbumin (OVA) (grade V; Sigma, St. Louis, MO, USA) with aluminum hydroxide (Thermo, Rockford, IL, USA) on days 1–3 and day 14, as described previously [14]. Normal saline, prednisolone, or acacetin is administered to mice on days 21–27. We also used a nebulizer (DeVilbiss Pulmo-Aide 5650D, USA) to administer 2% OVA by inhalation to challenged mice for 30 min on days 14, 17, 20, 23, and 27.

All mice were randomly divided into six groups (*n* = 12 per group): (1) normal control mice (N group) were sensitized and challenged with normal saline; (2) OVA control mice (OVA group) were sensitized and challenged with OVA and fed normal saline; (3) OVA-sensitized mice that were fed 25 mg/kg prednisolone (Sigma) were defined as positive control mice (P group); (4) OVA-sensitized mice that were fed 5, 10, or 20 mg/kg acacetin (Sigma) were named groups A5, A10, and A20, respectively.

### 2.3. Measurement and Analysis of AHR

On day 28, AHR was assessed with a whole-body plethysmograph (Buxco Electronics, Troy, NY, USA), as described previously [15]. Mice inhaled increasing doses of methacholine ranging from 0 to 40 mg/mL for 3 min each while inside the whole-body plethysmograph, and AHR was recorded and assayed as an enhanced pause (Penh).

### 2.4. Harvesting Supernatant and Counting Cells in Bronchoalveolar Lavage Fluid

Mice were anesthetized and sacrificed on day 29. Each animal's trachea was cannulated, and the lumen and lungs were flushed three times with 1 mL normal saline, defined as bronchoalveolar lavage fluid (BALF). The BALF was centrifuged, and cells were collected and stained with Liu stain solution to calculate the total cells and the percentage of eosinophils in each sample. After centrifugation, the BALF supernatants were collected, and cytokine and chemokine levels were measured.

### 2.5. Serum Collection and Splenocyte Cultures

After blood samples were centrifuged at 6000 rpm for 5 min at 4°C, the serum was collected, and OVA-specific antibody levels were measured. Splenocytes were prepared as previously described [16]. Splenocytes (5 × 10^6^ cells/mL) were cultured in RPMI 1640 medium (Invitrogen-Gibco, Paisley, Scotland) containing 10% fetal bovine serum (Biological Industries, Haemek, Israel), 100 U/mL penicillin/streptomycin, and 100 *μ*g/mL OVA for 5 days. Then, the culture supernatants were collected to assay cytokine concentrations.

### 2.6. Enzyme-Linked Immunosorbent Assay

The BALFs and splenocyte culture supernatants were measured using ELISA kits specific for IL-4, IL-6, IL-8, IL-13, eotaxin-1, tumor necrosis factor-*α* (TNF-*α*), intercellular adhesion molecule 1 (ICAM-1), prostaglandin E_2_ (PGE_2_) (R&D Systems, Minneapolis, MN, USA), and IL-5 (BD Biosciences, San Diego, CA, USA), as previously described [14, 17]. Serum OVA-specific antibody levels were measured using an ELISA kit specific for IgG1, IgG2a, and IgE (BD Biosciences), as previously described [16, 17]. Using a standard curve from pooled serum, we measured the units of OVA-specific IgG1 and OVA-IgG2a according to OD_450_ readings. To measure OVA-IgE levels, serum was diluted 5-fold and directly measured by OD_450_.

### 2.7. Lung Histology

Lungs were fixed with formalin, embedded in paraffin, and sliced into 6 *μ*m sections. The slides were stained with periodic acid-Schiff (PAS) stain using a PAS system (Sigma) or stained with hematoxylin and eosin (HE), as previously described [14, 18].

### 2.8. Western Blot Analysis

Proteins (10 *μ*g each) were separated on 10% SDS polyacrylamide gels, and the gel was transferred onto polyvinylidene fluoride membranes (Millipore, Billerica, MA, USA), as previously described [19]. The membranes were incubated with antibodies, including antibodies raised against COX-2 (Santa Cruz, CA, USA) and *β*-actin (Sigma), overnight at 4°C. Next, the membranes were washed and incubated with secondary antibodies for 1 h at room temperature, incubated with Luminol/Enhancer Solution (Millipore), and exposed using the BioSpectrum 600 system (UVP, Upland, CA, USA).

### 2.9. Immunohistochemistry

Lung sections were stained as described elsewhere [14]. Slide-mounted lung tissue was incubated with anti-COX-2 antibody overnight at 4°C. The slide was washed and incubated with secondary antibodies for 30 min at room temperature, and DAB substrate was added and observed with a light microscope.

### 2.10. Culture and Acacetin Treatment of BEAS-2B Cells

BEAS-2B human bronchial epithelial cells were cultured in DMEM/F12 medium (Invitrogen-Gibco) in 24-well plates. BEAS-2B cells (1 × 10^6^ cells/mL) were pretreated for 1 h with different concentrations of acacetin (3–100 *μ*M, dissolved in ≤0.1% DMSO). Next, cells were cultured with 5 ng/mL TNF-*α* for 24 h, or with 5 ng/mL TNF-*α* and 20 ng/mL IL-4 for 48 h. The supernatants were collected and measured using ELISA kits.

### 2.11. Cell-Cell Adhesion Assay

BEAS-2B cells (2 × 10^6^ cells/mL) were pretreated with a range of concentrations of acacetin for 1 h, then incubated with 5 ng/mL TNF-*α*, and cultured in 6-well plates for 24 h. Human differentiated eosinophilic HL-60 cells were treated with calcein-AM solution (Sigma) for 30 min at 37°C. HL-60 cells were washed and added to BEAS-2B cells for 1 h at 37°C. Finally, HL-60 cells adhered to BEAS-2B cells were observed and measured using fluorescence microscopy (Olympus, Tokyo, Japan).

### 2.12. Statistical Analysis

All results were estimated using one-way ANOVA. Data are presented as mean ± SEM, and *P* < 0.05 was considered statistically significant.

## 3. Results

### 3.1. Acacetin Reduced AHR in OVA-Sensitized Mice

**Figure 1:**
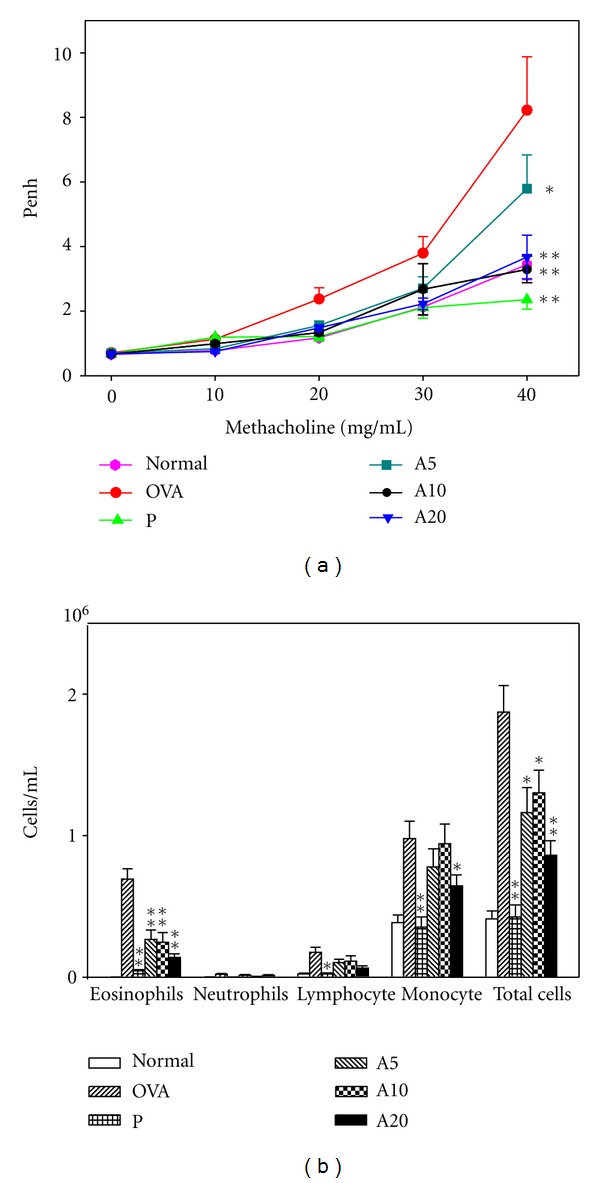
The effect of acacetin on AHR and cell counts in BALF. (a) After mice inhaled varying doses of methacholine (10–40 mg/mL), AHR was measured and is shown as Penh values. (b) Both total cell number and different cell counts were calculated in BALF. Data are presented as means ± SEM. *P < 0.05 compared with the OVA group. **P < 0.01 compared with the OVA group.

OVA-sensitized, asthmatic mice were fed acacetin once daily from days 21 to 27. AHR was measured on the next day after the last OVA challenge (day 28), and AHR was assessed as the Penh value after inhalation of increasing doses of methacholine (Figure 1(a)). After treatment with 10–30 mg/mL methacholine, Penh values of the OVA group did not differ significantly from those of mice fed varying doses of acacetin. However, after treatment with 40 mg/mL methacholine, OVA-sensitized mice fed varying doses of acacetin (A5, A10, and A20 groups) exhibited significantly reduced Penh values compared with the OVA group (8.22 ± 1.65) (A5, 5.79 ± 1.05, *P* = 0.09; A10, 3.29 ± 0.41, *P* < 0.01; A20, 3.28 ± 0.67, *P* < 0.01). OVA-sensitized mice that ingested prednisolone (P group) also exhibited significantly reduced Penh values (2.36 ± 0.29, *P* < 0.01). These data indicate that acacetin suppresses AHR in asthmatic mice.

### 3.2. Effect of Acacetin on Cellular Changes in BALF

After the animals were sacrificed, BALF was collected, and we calculated the numbers of total cells, monocytes, eosinophils, neutrophils, and lymphocytes (Figure 1(b)). OVA-sensitized mice exhibited significantly greater numbers of eosinophils and total cells compared with the N group. However, asthmatic mice that ingested acacetin (A5, A10, A20 groups) or prednisolone (P group) exhibited significantly reduced numbers of eosinophils and total cells compared with the OVA group. 

### 3.3. Effect of Acacetin on Chemokine and Th2-Associated Cytokine Levels in BALF

**Figure 2:**
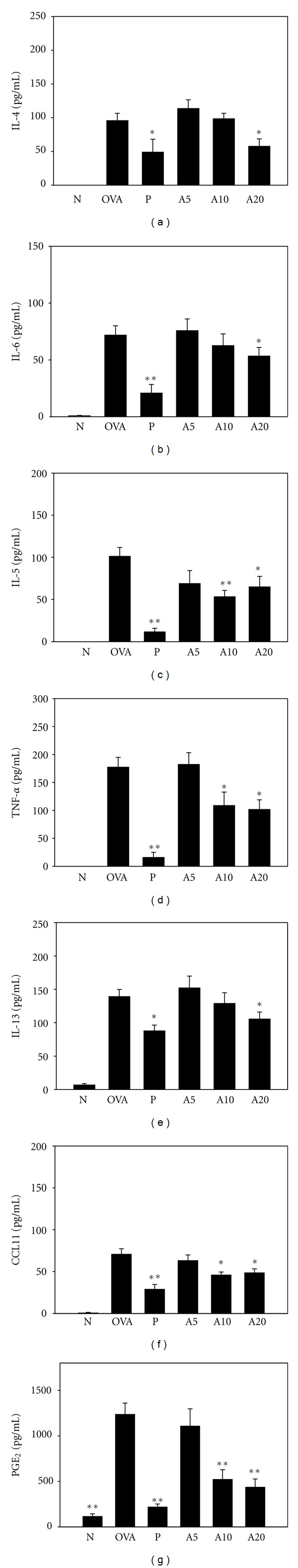
Acacetin reduced the levels of cytokines and chemokines in BALF. The concentrations of IL-4 (a), IL-5 (b), IL-13 (c), IL-6 (d), TNF-*α* (e), eotaxin-1 (f), and PGE_2_ (g) were measured by ELISA. Data are presented as means ± SEM. *P < 0.05 compared with the OVA group. **P < 0.01 compared with the OVA group.

To investigate the immunomodulatory effects of acacetin, we examined the levels of Th2 cytokines (IL-4, IL-5, IL-13), eotaxin-1 (also known as CCL11), and proinflammatory cytokines (TNF-*α*, IL-6) in BALF (Figure 2). ELISA analysis indicated that OVA-sensitized mice exhibited significantly greater IL-4, IL-5, and IL-13 levels compared with the N group. Treated with higher-dose acacetin (A20 groups) or prednisolone (P group), IL-4 and IL-13 were significantly reduced compared with the OVA group. In addition, A10 and A20 groups exhibited significantly suppressed levels of IL-5, IL-6, TNF-*α*, and eotaxin-1 in BALF. However, the IL-4, IL-5, and IL-13 levels of the A20 group did not differ significantly from those mice fed prednisolone.

### 3.4. Effect of Acacetin on Eosinophil Infiltration and Goblet Cell Hyperplasia in Lung

**Figure 3:**
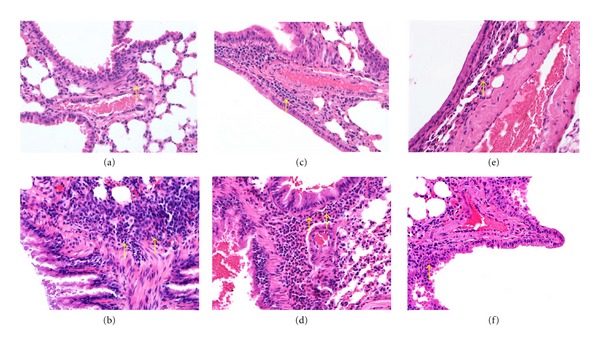
The effects of acacetin on eosinophil infiltration in lung tissue. Hematoxylin and eosin staining of lung tissue from normal (a), OVA (b), prednisolone (c), A5 (d), A10 (e), and A20 (f) groups (400x magnification). Eosinophils are indicated by arrows.

**Figure 4:**
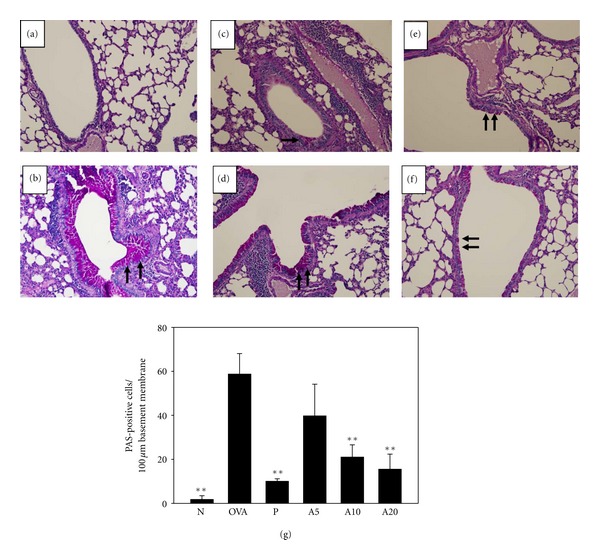
The effects of acacetin on goblet cell hyperplasia in lung tissue. Lung sections were stained with PAS stain to analyze goblet cell hyperplasia for normal (a), OVA (b), prednisolone (c), A5 (d), A10 (e), and A20 (f) groups (200x magnification). Goblet cells are indicated by arrows. Graphs represent the number of PAS-positive cells per 100 *μ*m of basement membrane (g) and were expressed as means ± SEM. **P < 0.01 compared with OVA control mice.

Staining of lung sections with HE revealed that more eosinophils had infiltrated between the trachea and blood vessels in OVA-sensitized mice than in normal control mice (Figure 3). In asthmatic mice fed with acacetin, especially the A10 and A20 groups, eosinophil infiltration was decreased significantly in the lung. In addition, PAS staining revealed a proliferation of goblet cell hyperplasia in the airways of asthmatic mice (Figure 4). Orally administered acacetin or prednisolone suppressed goblet cell hyperplasia. In addition, treatment with 20 mg/kg acacetin, goblet cell hyperplasia did not differ significantly compared with P group.

### 3.5. Acacetin Downregulated the COX-2/PGE_**2**_ Pathway

**Figure 5:**
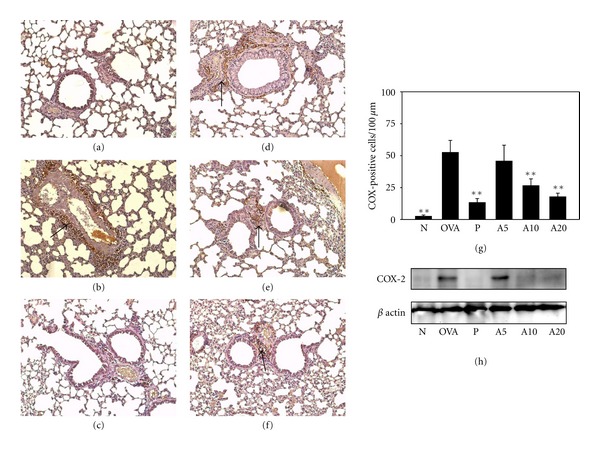
Acacetin reduced COX-2 expression in the lungs. COX-2 expression was analyzed by IHC staining (*brown*, indicated by arrows) in normal (a), OVA (b), prednisolone (c), A5 (d), A10 (e), and A20 (f) groups (100x magnification). Results were expressed as the number of COX-positive cells per 100 *μ*m (g). COX-2 protein levels were detected by Western blots in lung tissue (h); *β*-actin expression was used as an internal control. Data are presented as means ± SEM. **P < 0.01 compared with the OVA group.

Acacetin reduced the proinflammatory cytokine TNF-*α* and IL-6 content of BALF in asthmatic mice. We thought that acacetin might downregulate the COX-2/PGE_2_ lung inflammation pathway. When IHC staining was used to examine COX-2 protein distribution in the lungs, we observed almost no COX-2 expression in lung tissue from the N group (Figure 5). However, more COX-2 was detected in OVA-sensitized mice. Treatment with acacetin or prednisolone reduced COX-2 protein distribution in the lungs. Western blotting revealed a significant reduction of COX-2 expression in mice treated with prednisolone or acacetin in a dose-dependent manner. In addition, A20-inhibited COX-2 expression did not significantly differe compared with the P group. PGE_2_ levels in BALF were increased in the OVA group (1237.11 ± 123.64 pg/mL) (Figure 2). However, oral administration of prednisolone or acacetin significantly suppressed PGE_2_ levels in the P, A5, A10, and A20 groups compared with the OVA group. 

### 3.6. Effect of Acacetin on OVA-Specific Antibodies in Serum and Cytokine Levels in Splenocyte Culture Supernatant

**Figure 6:**
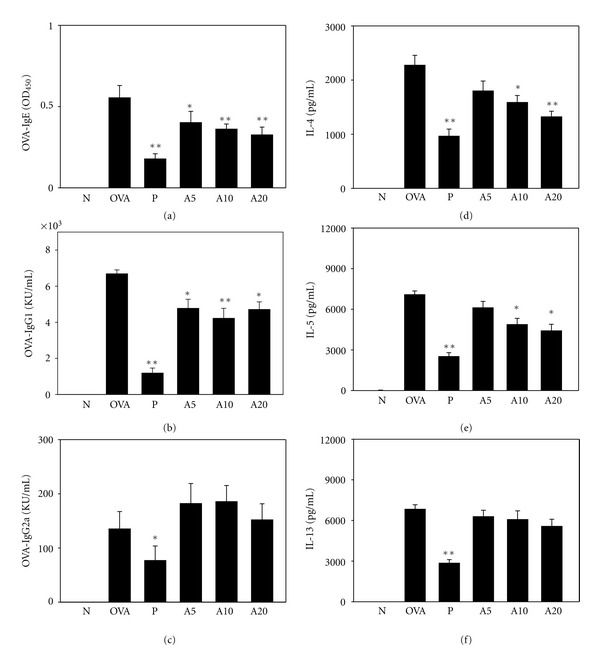
Acacetin affected the levels of OVA-specific antibodies in serum, as well as the levels of cytokines produced by OVA-activated splenocytes, including OVA-IgE (a), OVA-IgG1 (b), OVA-IgG2a (c), IL-4 (d), IL-5 (e), and IL-13 (f). Data are presented as means ± SEM. *P < 0.05 compared with the OVA group. **P < 0.01 compared with the OVA group.

OVA-specific IgE, IgG1, and IgG2a antibody levels were significantly increased in OVA-sensitized mice compared with normal control mice (Figure 6). Acacetin treatment significantly reduced OVA-IgE and OVA-IgG1 levels but did not significantly increase OVA-IgG2a. In splenocyte culture supernatant, IL-4 and IL-5 levels were significantly lower in OVA-sensitized mice fed with acacetin (A10 and A20 groups) than in the OVA group; however, there was no reduction of IL-13 level.

### 3.7. Suppression of Proinflammatory Cytokine and Chemokine Expression by Acacetin in Activated BEAS-2B Cells

**Figure 7:**
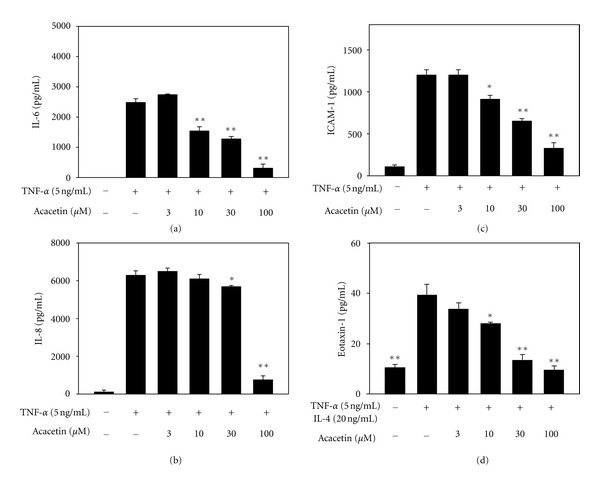
Acacetin affects cytokine and chemokine levels in BEAS-2B cells. The levels of IL-6 (a), IL-8 (b), ICAM-1 (c), and eotaxin-1 (d) were measured by ELISA. Data are presented as means ± SEM. *P < 0.05 compared with the activated control group. **P < 0.01 compared with the activated control group.

Bronchial epithelial cells release chemokines and proinflammatory cytokines to induce an inflammatory response and eosinophil migration in asthma patients [20]. BEAS-2B cells were stimulated with TNF-*α* to evaluate whether acacetin was able to suppress inflammatory mediators. The results demonstrated that acacetin did suppress levels of IL-6, IL-8, and ICAM-1. In addition, acacetin also significantly reduced eotaxin-1 when BEAS-2B cells were stimulated by TNF-*α* and IL-4 (Figure 7).

### 3.8. Inhibition of Eosinophil Adhesion to TNF-*α*-Activated BEAS-2B Cells by Acacetin

**Figure 8:**
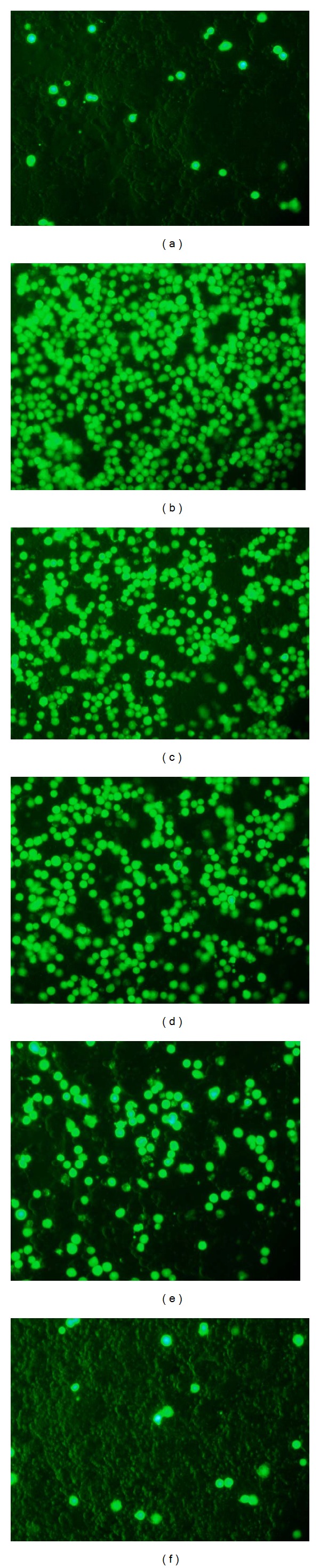
Acacetin suppressed eosinophil adhesion to BEAS-2B cells. HL-60 cells were treated with calcein-AM and added to BEAS-2B cells, and the results were observed using fluorescence microscopy. Note the adherence of HL-60 cells to normal (a) and TNF-*α*-activated BEAS-2B cells (b). Pretreatment of HL-60 cells with 3 *μ*M acacetin (c), 10 *μ*M acacetin (d), 30 *μ*M acacetin (e), and 100 *μ*M acacetin (f) reduced the adherence of HL-60 cells to BEAS-2B cells in a dose-dependent manner.

Inflammatory BEAS-2B cells may express more ICAM-1 to increase eosinophil adhesion. Therefore, we investigated whether acacetin was able to suppress eosinophilic adherence to TNF-*α*-activated BEAS-2B cells. HL-60 cells were stained with calcein-AM and added to TNF-*α*-activated BEAS-2B cells. A greater number of HL-60 cells adhered to TNF-*α*-activated BEAS-2B cells than nonactivated BEAS-2B cells. In addition, pretreatment with acacetin reduced the adhesion of HL-60 cells to BEAS-2B cells (Figure 8).

## 4. Discussion

The pathophysiology of asthma includes AHR, eosinophil infiltration of lung tissue, and tracheal smooth muscle contraction [2]. In addition, tracheal goblet cell hyperplasia causes the oversecretion of mucus, which can lead to suffocation or death [5]. Steroids are often used in the clinical setting to treat asthma, and theophylline, anti-histamine drugs, and COX-2 inhibitors also are used to treat or prevent asthma attacks [21–23]. However, eastern and western medicine are still searching for other alternative asthma therapies [24].

In traditional Chinese medicine and dietary supplements, several types of single agents, complex herbal formulas, or pure compounds have been shown to improve asthma symptoms, including CVT-E002, Danggui Buxue Tang, *Gynostemma pentaphyllum*, viscolin, curcumin, and lycopene [15, 18, 25–29]. In this paper, we have investigated whether acacetin is able to attenuate asthma symptoms in a mouse model. We also used inflammatory airway epithelial cells to evaluate the regulation of eosinophil chemotaxis and its mitigation in the lung. We observed that orally administered acacetin was able to inhibit AHR, reduce eosinophil infiltration of lung tissue, and suppress goblet cell hyperplasia in the trachea. Treatment with acacetin decreased the levels of Th2-associated cytokines, eotaxin-1, and proinflammatory cytokines in BALF. We also observed reduced expression of the inflammatory mediator COX-2 in lung tissue. In addition, acacetin suppressed the levels of proinflammatory cytokines and chemokines (including IL-8 and eotaxin-1) and reduced the ability of eosinophils to adhere to tracheal epithelial cells. Hence, we hypothesized that acacetin might improve pathological and inflammatory airway symptoms in asthmatic mice.

Previous studies have found that excessive secretion of Th2 cytokines, including IL-4, IL-5, and IL-13, is able to aggravate pathological asthma symptoms [7]. IL-4 activated B cells and induced B cells to produce IgE [30]. When allergens combine with IgE and mast cells, mast cells are activated and release histamine to cause severe allergic reactions. Lycopene and dehydroepiandrosterone reduce the level of IL-4 in BALF and OVA-IgE in serum of OVA- sensitized mice [14, 28]. The level of IgE is able to decrease in IL-4 knockout asthmatic mice [31]. Omalizumab, is a humanized monoclonal anti-IgE antibody and suppresses IgE levels of serum and IgE receptor expression on mast cells in asthma patients [32]. Hence, acacetin significantly inhibits OVA-IgE production in serum through the suppression of IL-4.

Eosinophils also play an important role in asthma [33]. There are more activated eosinophils in the lungs of asthmatic patients, and those cells release more inflammatory mediators (e.g., eosinophil cationic protein) to aggravate the lungs of asthmatic patients [34]. IL-5 is a very important factor that induces the differentiation of bone marrow cells into active eosinophils [35]. In asthma, eotaxin-1 induces activated eosinophils to migrate into the lungs. Our experimental results indicate that acacetin decreases IL-5 and eotaxin-1 levels, which in turn reduced eosinophil infiltration into lung tissue. A previous study found that activated tracheal epithelial cells expressed the adhesion molecule, ICAM-1, to increase eosinophil adherence and migration into the lungs [5]. The present results demonstrate that acacetin not only attenuates asthma symptoms by reducing IL-5 and decreasing eotoxin-1 levels for eosinophil activation but also reduces ICAM-1 expression in tracheal epithelial cells to reduce the ability of eosinophils to adhere to tracheal epithelial cells and migrate into the lungs.

AHR is also an important pathological symptom in asthma patients, and IL-13 may enhance AHR [3]. Phosphoinositide 3-kinase inhibitor can attenuate IL-13-induced AHR in asthmatic mice [36]. Although orally administered acacetin did suppress IL-13 levels in BALF from OVA-sensitized asthmatic mice, it did not inhibit IL-13 production in splenocyte cultures. In this experimental model, believe that acacetin improves the local immune response of the lungs to AHR but does not modulate IL-13 levels in the systemic immune response.

Previous studies found that IL-4 and IL-13 increased tracheal goblet cell hyperplasia [3]. We thought that treatment with acacetin significantly reduced goblet cell hyperplasia of the airway by suppressing the levels of IL-4 and IL-13 in BALF. In recent years, treatment with anti-IL-4 or anti-IL-13 monoclonal antibodies significantly inhibited AHR, goblet cell hyperplasia, and collagen deposition in asthmatic mice [37, 38]. During an asthma attack, in addition to tracheal contraction, goblet cells secrete more mucus, which causes obstruction and difficulty breathing through narrowed airways. Tracheal goblet cells secrete mucus mixed with mucin, and the mucin affects and enhances the elasticity and adhesion of the mucus [2]. MUC5AC is the major mucin in airways of asthma patients [39]. We observed that acacetin reduces MUC5AC gene expression in lung tissue (data not shown). Therefore, our results suggest that orally administered acacetin might attenuate airway inflammation, eosinophil infiltration, and goblet cell hyperplasia by suppressing levels of Th2-associated cytokines in OVA-sensitized mice. In addition, we found mice were sensitized and challenged with normal saline, and fed acacetin does not affect AHR and eosinophil infiltration in physiological functions of respiratory tract (data not shown). 

Airway inflammation can cause lung tissue damage or fibrosis in asthma patients [40]. Therefore, reducing airway inflammation may improve symptoms of asthma. The previous results suggest that acacetin inhibits proinflammatory cytokines and COX-2 in LPS-activated macrophages [8]. A COX-2 inhibitor is currently used to treat asthma in the clinical setting. It would be interesting to determine whether acacetin, which inhibits COX-2 expression in lung tissue, would be an effective treatment for asthma. 

Prednisolone is a strong immunosuppressive drug to inhibit Th1 and Th2 immune response. Hence, all cytokines and antibodies significantly are suppressed. Acacetin only inhibited part of the Th2-associated cytokines and antibodies did not inhibit Th1-associated antibody OVA-IgG2a. The results suggest that acacetin is not an immunosuppressant, and acacetin is the good natural product that can regulate the immune response of asthma patients. In the future, we plan to evaluate the clinical effect of acacetin for asthma patients.

## 5. Conclusion

In conclusion, our results demonstrate that dietary acacetin is able to suppress AHR, airway inflammation in OVA-sensitized mice. Our data support the proposition that acacetin not only attenuates asthma symptoms by reducing Th2-associated cytokines but also reduces eotaxin-1 to reduce eosinophil infiltration of lungs. Hence, acacetin has the effects of anti-inflammation and antiasthma by modulating balance of the Th1/Th2 cytokines. It is a potential therapeutic nature production for improving AHR and airway inflammation in murine model of asthma.
